# Correction to: Antiplatelet therapy in patients with atrial fibrillation: a systematic review and meta-analysis of randomized trials

**DOI:** 10.1093/ehjcvp/pvaf013

**Published:** 2025-04-08

Correction to: Alexander P Benz, Isabelle Johansson, Willem J M Dewilde, Renato D Lopes, Roxana Mehran, Samantha Sartori, Nikolaus Sarafoff, Satoshi Yasuda, William F McIntyre, Jeff S Healey, Ashkan Shoamanesh, John W Eikelboom, Stuart J Connolly, Antiplatelet therapy in patients with atrial fibrillation: a systematic review and meta-analysis of randomized trials, *European Heart Journal - Cardiovascular Pharmacotherapy*, Volume 8, Issue 7, November 2022, Pages 648–659, https://doi.org/10.1093/ehjcvp/pvab044

In the original version of Supplemental Figure II (panel B) and Supplemental Figure V (panel B) some studies were erroneously included in the wrong category. This resulted in incorrect pooled estimates for the effect of antiplatelet therapy on all-cause stroke and ischemic stroke in patients with atrial fibrillation treated with background oral anticoagulation in the subgroups of patients without and with additional background antiplatelet therapy.

The authors have corrected this error and provided updated Forest plots. The results are materially unchanged.

These details have been corrected only in this correction notice to preserve the published version of record.

